# Knowledge of undergraduate dental students regarding management of caries lesions

**DOI:** 10.1038/s41405-022-00101-z

**Published:** 2022-04-01

**Authors:** Anahita Jablonski-Momeni, Heike Korbmacher-Steiner, Alexa Temming, Pia Wernke, Monika Heinzel-Gutenbrunner, Rainer Haak, Felix Krause

**Affiliations:** 1 Department of Orthodontics, Medical Faculty, Dental School, Philipps-University Marburg, Marburg, Germany; 2 MH Statistik, Marburg, Germany; 3 Department of Cariology, Endodontology and Periodontology, University of Leipzig, Leipzig, Germany; 4 Clinic for Operative Dentistry, Periodontology and Preventive Dentistry, University Hospital RWTH Aachen, Aachen, Germany

**Keywords:** Dental clinical teaching, Dental foundation training

## Abstract

**Objectives:**

Understanding of lifelong control of disease processes associated with caries and its management is an essential part of dental education. This study evaluated the dental students’ knowledge of caries diagnosis and management using the International Caries Classification and Management System (ICCMS).

**Methods:**

A survey was conducted among undergraduate dental students at two dental schools, attending the sixth (centre 1) and seventh semester (centre 2), respectively. Medical histories, clinical images and radiographs of 12 patients were compiled as anonymous cases. For each case, a specific lesion was to be assessed. In addition, the students should determine the patient’s caries risk and select a treatment option. An expert report (consensus decision) was used as the reference standard. For statistical analysis, kappa statistics and binomial tests were used.

**Results:**

A total of 46 students participated in this study. The percentage of agreement of responses to the reference was: centre 1: 40.7–51.3%, centre 2: 57.9–67.9%. The corresponding Kappa values were: centre 1: 0.073–0.175, centre 2: 0.315–0.432. Overall, students tended to underestimate the codes compared to the reference standard (*p* < 0.001).

**Conclusion:**

Introducing systematic content about caries diagnosis and management such as ICDAS and ICCMS in the learning objectives of undergraduate dental students can be proposed. However, in order to improve diagnosis and enable a more reliable choice of treatment options, attention should also be given to the way they are didactically taught.

## Introduction

With the publication of ‘The Graduating European Dentist’ in 2017, the Association for Dental Education in Europe (ADEE) provided a new approach to reflect the best academic practices for European undergraduate dental education [1]. The new series of documents placed greater emphasis on key curriculum components such as patient safety, working as a team, and patient-centred care. The documents also provided guidance on teaching and learning methods and assessments. Already in 2010, the ADEE had developed a catalogue of requirements for the European teaching curriculum and had defined core competencies for dentists graduating in Europe [2]. On the topic of ‘clinical information gathering’, the catalogue states: ‘On graduation, a dentist must be competent at:… Identifying the location, extent and degree of activity of dental caries, tooth wear, and other structural or traumatic anomalies and the reason for their occurrence…’. Moreover, a graduate dentist must be able to ‘select and prioritise treatment options that are sensitive to each patient’s individual needs, goals and values, compatible with contemporary therapy and congruent with human rights, a comprehensive oral health care philosophy, and healthcare economics’ [3]. With regard to caries detection, a graduate dentist must be able to ‘apply the scientific knowledge base relating to: The aetiology, pathology, diagnosis and management of oral diseases and disorders including (but not exclusively): i) caries, ii) tooth surface loss, …’ [4].

As a national effort, in Germany, a National Competence Based Catalogue of Learning Objectives for Undergraduate Dental Education was created in 2015 [5], which aimed to determine the profile of dentists after graduation. Corresponding work packages also dealt with the topic of ‘hard tooth structure defects’ that includes adequate detection, diagnosis and management of caries.

The International Caries Detection and Assessment System (ICDAS) enables a standardised visual assessment of caries at the enamel and dentine stages [6]. Accordingly, caries management can be planned and implemented in the early stages of caries development. As a continuation of the ICDAS and to present a standardised procedure for caries management, the International Caries Classification and Management System (ICCMS) was developed [7–9]. Therein, patient-related parameters were combined with clinical findings on the extent and activity of caries in order to plan and perform individual treatments. This system was designed to be flexible to the merged ICDAS scores because some scores required similar management. Regarding decision making, the ICCMS advocates the prevention of new carious lesions and controlling the progression of existing lesions. Thus, it preserves the structure of the tooth with non-operative care of lesions at early stages and tooth-preserving operative care for severe lesions [10].

The visual caries detection system, ICDAS, has already been implemented in the undergraduate dental curriculum in some German dental faculties [11, 12]. Following these efforts and the latest European approaches in dental education, it was proposed to introduce the ICCMS as a standardised system for caries diagnosis and management into the undergraduate curriculum. In preparation for this goal, the aim of the present study was to investigate the knowledge of undergraduate dental students regarding the staging of caries lesions, caries risk assessment and treatment options of caries lesions using a questionnaire.

## Methods

The study was conducted as a survey using a written questionnaire among dental students at two German dental schools. The project was approved by the ethics committee of the Medical Faculty of Philipps-University Marburg (Centre 1, Ref No. 112/18) and the ethics committee of the Medical Faculty of the University of Leipzig (Centre 2: Ref No. 398/18-lk).

### Subjects

The dental students were asked to voluntarily participate in the study. Students from centre 1 were in the sixth semester (*n* = 27), whereas students from centre 2 were in the seventh semester (*n* = 32). The inclusion criterion was participation in lectures and seminars in cariology. No further defined exclusion criteria were established. All students provided written informed consent to participate in the study.

Caries diagnosis was a part of the curriculum in both centres. In each centre, the lecturer was a member of the corresponding dental faculty with training and experience in the field of cariology as well as in ICDAS/ICCMS. The teaching content and material regarding caries diagnosis based on the ICDAS criteria were almost the same in both centres since both lecturers had compared and matched the teaching contents beforehand. Students in the sixth semester did not have any clinical experience but were already introduced to the ICDAS in corresponding lectures prior to the study. In detail, the training in the sixth semester consisted of a two-hour introductory lecture on ICDAS held in presence. Information about the background of ICDAS and the classification of codes was presented and exercises on selected images were performed and discussed during the session. Then, the students were introduced to the ICCMS webpage and had the task to complete the 60-min e-learning video on ICCMS core training: Element 2, which provides the caries classification systems (https://www.iccms-web.com/content/resources/elearning).

Students from centre 2 had already attended the theoretical course in their previous semester as described above. When the study was performed, the students were completing the clinical course in preventive and conservative dentistry and had therefore more clinical experience compared to the students in centre 1. During this clinical semester, the students from centre 2 used the ICDAS criteria in their patients and entered the findings in corresponding examination sheets. The findings were controlled by the clinical lecturer and were discussed. Each student examined four to five patients during the course.

### Structured questionnaire

The medical history of 12 patients aged 7–18 years who were treated in the dental school of centre 1 was compiled into anonymous cases. Each patient agreed to have their pictures used for teaching and study purposes. The cases were given to the students electronically, together with a questionnaire for viewing on a computer screen. The students completed the questionnaires anonymously over a period of 10 days. Questionnaires were then collected at each centre by a person not involved in the course of the study.

**Fig. 1:**
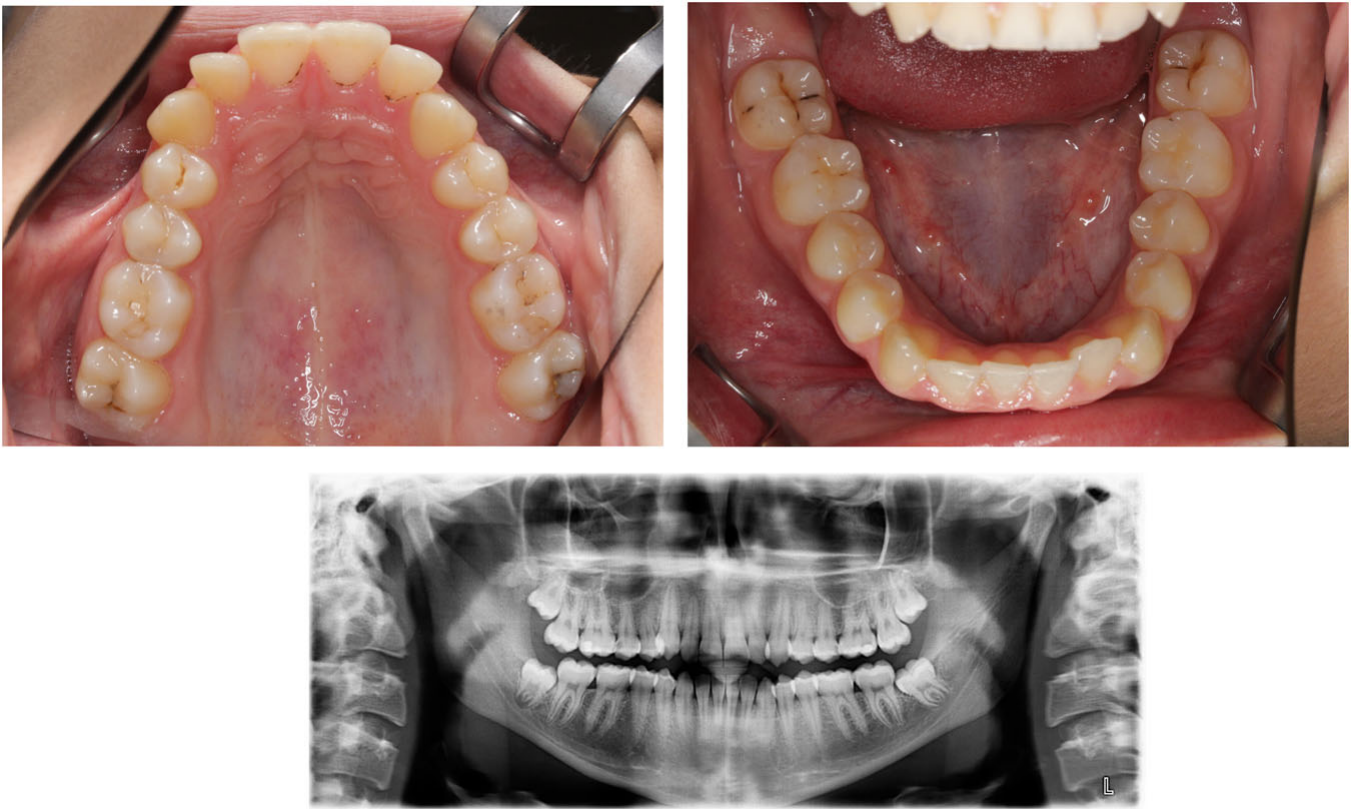
Example of a presented case: female with no general diseases and medication and early loss of tooth #55 due to caries that was treated with a space maintainer. She underwent no further orthodontic treatment. Her follow-up dental visits were irregular. The tooth to be inspected was #15.

In each case, the patient’s age, sex, and general medical and dental history were indicated. Relevant information was provided to indicate the caries risk of the patient. In addition, clinical and radiographic images were displayed. To create the clinical images, the teeth were dried for 5 s according to the ICDAS protocol prior to photography in each jaw. A representative case is shown in Fig. 1.

For each clinical case, a specific lesion was assessed. The area of interest was marked with arrows. Students were asked to assess the ICCMS code using clinical and radiographic images. First, a visual assessment needed to be performed based on the merged ICDAS criteria as follows [7].

(1) Sound tooth: no evidence of change in enamel translucency due to caries after plaque removal and air-drying. (2) Initial caries lesions (ICDAS 1-2): changes in enamel seen as a carious opacity or visible discolouration (white/brown spot) not consistent with the clinical appearance of sound enamel with no evidence of surface breakdown, no underlying dentine shadowing or cavitation. (3) Moderate caries lesions (ICDAS 3-4): white/brown spot lesion with localised micro-cavity/discontinuity, without visible dentine exposure and best seen after air-drying, or obvious discolouration of dentine visible through apparently intact or micro-cavitated enamel surface, which originated on the surface being evaluated. Often, such lesions are most easily visualised with the tooth surface wet. (4) Extensive caries lesions (ICDAS 5-6): obvious visible dentine cavity in opaque/discoloured enamel.

The corresponding radiographs needed to be evaluated based on the following radiological caries stages: code 0 = no radiolucency in the enamel or dentin; code 1 = radiolucency in the enamel up to the enamel-dentine junction; code 2 = radiolucency in the first 1/3 of dentine; code 3 = radiolucency reaching the middle one-third of dentine; and code 4 = radiolucency reaching the inner one-third of dentine up to the pulp.

Thereafter, students needed to choose a combined ICCMS code (ICDAS + radiographic findings) and mark the answers in the questionnaire by combining the visual and radiographic findings. The ICCMS codes were interpreted as sound tooth, initial carious lesion, moderate carious lesion, or extensive carious lesion. For each question, there was also a ‘no answer’ option, in case a student was unable to choose an answer. This was later defined in the statistical evaluation as ‘missing answers’.

The next step was to evaluate the caries risk (low, moderate, or high) for each patient case. The caries risk needed to be determined in accordance with the patient’s general medical and dental history. For this parameter and for the treatment options, students were advised to consider all dental findings shown on the clinical images and radiographs.

The treatment planning option for the marked tooth needed to be selected from the following list: Option 1 = basic preventive care (e.g., recommended homecare, including use of fluoride toothpaste [≥1000 ppm F^–^] twice per day, as well as clinical approaches such as motivational management and professional cleaning). Option 2 = basic preventive care and additional use of fluoride (e.g., recommend F^–^varnish application, F^–^mouth rinse, and higher efficacy fluoride toothpaste (≥1.450 ppm F^–^). Option 3 = basic preventive care and micro-invasive treatment (e.g., fissure sealing, infiltration, or regenerative treatment options). Option 4 = operative treatment in addition to preventive care.

### Determination of the reference standard

Before distributing the questionnaires to the participants, two experts from centres 1 and 2 answered the questions independently first. No extreme discrepancies between the responses were found. In case of a disagreement, the results were discussed and a consensus was reached.

### Statistical analysis

Statistical evaluation was carried out using the statistical programme SPSS Statistics for Windows, V 24 (IBM Corp. Armonk, NY).

The answers of the students in the preclinical year (third year) were compared with those of the students in the first clinical year (fourth year). The reference standard was based on the consensus decision of the two experts in both centres.

The agreement between the students’ responses and the reference was determined using Cohen’s kappa coefficients.

For discordant answers, the percentage of students’ responses to all variables (ICCMS combination code, caries risk, treatment option) were checked for over- or underestimation by means of binomial tests (*α* = 0.05).

## Results

A total of 96.7% of the students in the third year (26 out of 27, centre 1) and 62.5% of students in the fourth year (20 out of 32, centre 2) took part in the study. The numbers of male and female student participants from centre 1 were seven and 19, respectively, while four male and 16 female students took part from centre 2.

The number of missing values for combined ICCMS codes (caries stages + radiographic findings), caries risk assessment, and treatment options were comparable in both centres; 2.9% and 3.75% of the ICCMS codes, 2.9% and 1.67% of the caries risk assessment, and 1% and 1.67% of the treatment option data were missing from centre 1 and centre 2, respectively.

In general, the percentage concordance of students’ responses to the reference was higher in centre 2 than in centre 1 (Table 1). For answers that were discordant to the reference, students from centre 1 tended to underestimate the codes compared to the reference standard (Table 1). However, in centre 2, no significant difference was seen between under- and overestimation of the combined ICCMS codes.

**Table 1:** Concordance between the students’ performance and the reference standard and over- and underestimation of answers discordant with the reference.

Variable		Centre 1	Centre 2
		(6th semester)	(7th semester)
ICCMS combined code	All-over concordance	48.4%	67.9%
	Underestimation	73.0%	51.0%
	Overestimation	27.0%	49.0%
	*p* value*	<0.001	0.998
Caries risk	All-over concordance	51.3%	57.9%
	Underestimation	80.0%	92.0%
	Overestimation	20.0%	8.0%
	*p* value*	<0.001	<0.001
Treatment option	All-over concordance	40.7%	67.5%
	Underestimation	84.0%	77.0%
	Overestimation	16.0%	23.0%
	*p* value*	<0.001	<0.001

*p values refer to the difference between under- and overestimation.

Regarding different ICCMS stages, the agreement between students and the reference standard was: ICCMS initial lesions: centre 1: 36.0%, centre 2: 59.5% (both centres 46.7%); ICCMS moderate lesions: centre 1: 50.0%, centre 2: 69.0%, (both centres 58.7%) and ICCMS extensive lesions: centre 1: 50.0%, centre 2: 71.4% (both centres: 59.8%).

The mean simple kappa values for agreement of the response to the reference standard are shown in Table 2. A slight agreement [13] was found for the variables in centre 1, whereas a fair-to-moderate agreement was found in centre 2.

**Table 2:** Mean simple kappa values for concordance between the responses of students in each centre and the reference.

Variable	Centre 1 (6th semester)	Centre 2 (7th semester)
ICCMS combined code	0.175	0.432
Caries risk	0.178	0.315
Treatment option	0.073	0.361

Interpretation of *κ* (Landis and Koch) [13]: *κ* < 0.00 indicates poor agreement, *κ* = 0.00–0.20 indicates slight agreement, *κ* = 0.21–0.40 indicates fair agreement, *κ* = 0.41–0.60 indicates moderate agreement, *κ* = 0.61–0.80 indicates substantial agreement, and *κ* > 0.80 indicates almost perfect agreement.

## Discussion

In the present study, the students’ assessment of caries detection and diagnosis, caries risk, and caries management was recorded through a questionnaire. The assessment of students from centre 1 (without clinical experience) and centre 2 (with first clinical experience) was compared. The consensus of experts from both centres served as a reference standard.

The need for dental students to receive a systematic and profound education in cariology has long been recognised [14]. While caries detection and diagnosis are now established among undergraduate students with standardised systems such as ICDAS [15], the assessment of caries risk and caries management is still not the subject of a systematic approach, at least not in the centres where the present study was conducted.

To support a shift towards a more preventive and outcome-oriented philosophy, the ICCMS was developed as a framework that synthesises risk assessment data with caries classifications [7]. The aim was to systematically support clinicians and educators in identifying patients at risk of caries and, wherever possible, treat them non-invasively. Where invasive treatment was required, the ICCMS provided guidance on how to maintain healthy tissue. In the ICCMS concept, the management of the lesions is related to the diagnosis of the individual lesions. For example, initial active lesions in general are managed with non-operative care whilst moderate/extensive lesions are in general managed operatively with tooth-preserving operative care. This is based on the individual risk management plan that includes the recall interval, the monitoring of the status of caries lesions and the reviewing of the patient behavioural change plan [7].

In the present study, the patient cases and related questions were adapted from ICCMS guidelines, taking into account four key elements: history, classification, decision making, and management. All information was presented in a simplified and concise manner, as appropriate, considering that the participating students had little or no experience in clinical dentistry. A limitation of the study is that information on dietary habits, use of fluorides, or oral hygiene was not provided. However, the importance of preventive measures and appropriate prophylactic measures that can be undertaken at home and in the context of regular dental follow-up visits will be addressed in further courses and lectures, both theoretically and practically. Therefore, the patient’s medical history and clinical findings were the most important information for determining the current caries risk. Students were instructed to assess the caries stages on the basis of visual intraoral and radiographical findings. Furthermore, students were instructed to synthesise the information collected and decide on an individualised treatment plan [16].

A more practical way of integrating the ICCMS guidelines into routine dental work was recently presented by the CariesCare International Charity [17]. The CariesCare practice guide is derived from the ICCMS and provides a structured update for dentists to help them achieve optimal caries care and results for their patients. An example of how a patient´s case can be assessed was presented by Beltrán et al. [18]. Our present study was conducted before the publications of Martignon et al. [17] and Beltrán et al. [18]. Therefore, we could not refer to their guidelines in our preparations.

The categories of caries were presented in the ICDAS codes and summarised in a condensed form for students. This was done because reproducibility values of ICDAS and ICDAS merged codes does not significantly differ between examiners [19]. The simplified nature of the criteria may increase its acceptance by undergraduate students when using such detailed criteria for caries detection. In another study it could be shown that undergraduate students were able to learn and use the two-digit ICDAS system, including each caries code (from 0 to 6) and the restoration codes appropriately after a short period of teaching [11, 12].

The examination of caries on the basis of clinical and radiographic images using the ICDAS criteria does not differ from the scoring of extracted teeth, as shown by Bottenberg et al. [20]. In addition, the high quality of digital photography offers dentists a clinical picture similar to that observed in person in clinics. Digital media are increasingly being used in dental education. Especially for caries detection, images of teeth are often used for training purposes. Jablonski-Momeni et al. concluded that images had good diagnostic performance, regardless of whether the teeth were dry or wet [12]. The use of images allows larger groups of participants and facilitates the conduct of multicentre studies such as ours. As a long-term consequence, the content and sequence of instruction can be standardised between different centres [12].

In the present study, students in the seventh semester showed better results in the assessment of caries stages, patient’s risk and caries management options. These students had typically more clinical experience than the participants in centre 1 who attended the sixth semester and did not work with patients yet. It was reported that the clinical experience of the examiners had no influence on the performance of caries assessment with a visual inspection [15, 21]. Jablonski-Momeni et al. [11] reported in another study that an additional theory lesson improved the reproducibility of responses by students who had just started learning the ICDAS method. In that study mean kappa values (weighted kappa) for intra- and inter-examiner reproducibility values were between 0.34 and 0.65. However, the kappa values of the two groups did not differ significantly (*p* > 0.05). Zandona et al. [22] investigated the use of ICDAS for severity and activity assessment in three different groups and found that the differences in agreement between students, graduates and teachers were not statistically significant. In their study, mean kappa values for faculty members ranged between 0.52 and 0.81, comparable to the kappa values for graduates (0.54–0.84). Kappa values of the undergraduates were between 0.59 and 0.79. The authors concluded that previous clinical experience did not play a significant role in learning the ICDAS. Others, however, have shown that students with lower clinical exposure, in contrast to graduates, had difficulties in assessing caries management, especially in the early stages of caries [23].

Our findings showed that in cases of non-agreement between students’ responses and the reference, students’ decisions tended to underestimate almost all variables. Possibly the students without lots of experience have the fear to overestimate carious lesions and would rather underestimate the findings. Other studies showed that students of dentistry in their final year of study tend to choose invasive treatment options with a tendency to over-treatment [24, 25]. Experienced clinicians base their decisions on past experiences, whereas less experienced clinicians use ‘manuals’ as guidance for decision making with regard to the diagnosis and adequate treatment planning [26–28].

Although assessing the activity of a lesion is an important part of the process of diagnosing caries, it was not included in our questionnaire. This was mainly because the assessment of caries activity is usually based on several clinical variables, and no single factor is normally valid exclusively for the definition of caries activity [29]. Certainly, specific indicators, such as the location of the lesion, presence of plaque, and tactile characteristics, such as surface roughness or bleeding tendency of gingiva, can be used as predictors for caries activity assessment [30–32]. In our case presentations, such variables could not be presented in an adequate and informative way and the teeth were cleaned before the images were taken, so the presence of plaque could not be assessed. These problems may have led to an underestimation of the need for treatment. Nevertheless, emphasis must be placed on training students in activity assessment in a clinical setting.

In contrast to that in the study by Bervian et al. [24], wherein only radiographs and no clinical pictures were provided, the case presentation in our study was closer to that seen in daily dental practice. In the study by Noguiera et al. [21], radiographic images were used only selectively, as the rest of the assessment was based on the clinical situation. However, in the present study, each patient case was presented with radiographic images, and a combination of visual and radiographic images was requested according to the findings of the ICCMS for caries assessment.

Our students had the task to determine the caries stage of only one tooth per patient and then decide on caries risk and treatment recommendations for each case. The rationale was to keep the questionnaire and data collection as clear and straightforward as possible to be suitable for undergraduates, since this was the first time such a survey had been conducted among our students. In further studies, the feasibility and outcome of scoring more teeth (or full dentition) of a patient can be evaluated. In any case, the presentation of relevant details of the patient’s history with the clinical and radiographic images gave the participants a more comprehensive view of the patient’s case. The options listed in the questionnaire corresponded to treatment options that are usually relevant to the centres where the study was conducted. To date, only a few results are available regarding students’ decisions of treatment options in primary teeth. Bussaneli et al. reported that the examiner’s experience influenced the treatment decision of occlusal initial lesions in primary teeth and that experienced dentists had a less invasive approach than undergraduate students [25].

This study was conducted in two dental schools. Firstly, the reference experts in the two centres have worked together in the field of cariology and caries detection for several years and both have a comparable background in this area [33]. Therefore, and due to the coordination of the content of the slides in the lectures, the teaching content on caries diagnostics and treatment planning was almost identical in both centres. Secondly, by including two dental schools, a higher number of participants could be achieved. However, the course content takes place in centre 1 during the second half of the third academic year (sixth semester) and in centre 2 during the first half of the fourth academic year (seventh semester). In both centres, the eligibility of students from other semesters could not be granted because of the tight schedule and other reasons.

The response rate differed between the two centres (96.7% in centre 1 and 62.5% in centre 2). This may be explained as follows: participation in the study was voluntary and without incentives or special benefits for the students. Furthermore, participation was not relevant to passing the course. There was only one reminder to fill out the questionnaires within 10 days. Since the students in centre 2 were in the clinical year, they were assumed to have a tighter schedule than that of students in centre 1. However, the total number of participants did not largely differ between the centres.

In the present study, follow-ups were not planned or performed due to the reasons discussed above. This may be a limitation of this study and should be addressed in future projects. In another study, Jablonski-Momeni et al. showed that fifth-year dental students with previous experience in the ICDAS system could detect initial lesions in digital images significantly better than students in the third year of their dental education [12].

## Conclusion

The key competencies required for dentists are described in the European Core Curriculum, and three major competencies were identified: risk assessment, diagnosis, and synthesis [34]. This questionnaire-based study showed a low level of agreement between undergraduate students and experienced dentists in these competencies. These skills could be improved by integrating systematic procedures for caries detection and management into the clinical dental curriculum. In addition, the requirements for the European catalogue of learning objectives would be better met in the long term. In dental schools where the ICDAS is already integrated into the curriculum, the introduction of ICCMS can be proposed as a concept in the learning objectives of dental students. In addition, more attention should be paid in future to the didactic teaching of ICDAS and ICCMS.

## Data Availability

The dataset generated and analysed during the current study is available from the corresponding author on reasonable request.
